# Novel Electrochemical Aptasensor Based on Iron–Cobalt-Doped Magnetic Carbon and cDNA-Polyacrylic Acid for the Determination of Aflatoxin B1 in Peanuts

**DOI:** 10.3390/s26144348

**Published:** 2026-07-09

**Authors:** Zhongyu Li, Zili Xia, Dongdong Chen, Yang Han, Heng Zhang, Xia Sun, Wenping Zhao

**Affiliations:** 1 School of Agricultural Engineering and Food Science, Shandong University of Technology, No. 266 Xincun Xilu, Zibo 255049, China; 17860531699@163.com (Z.L.); 18326109893@163.com (Z.X.); 19862008815@163.com (D.C.); 15695441875@163.com (Y.H.); 15865955115@163.com (H.Z.);; 2 Shandong Provincial Engineering Research Center of Vegetable Safety and Quality Traceability, No. 266 Xincun Xilu, Zibo 255049, China; 3 Zibo City Key Laboratory of Agricultural Product Safety Traceability, No. 266 Xincun Xilu, Zibo 255049, China

**Keywords:** electrochemical aptasensor, aflatoxin B1, rapid determination, peanut, polyacrylic acid

## Abstract

The presence of aflatoxin B1 (AFB1) is ubiquitous in the environment, and it is considered one of the most powerful natural carcinogenic substances. In this study, a highly sensitive electrochemical aptasensor was designed to detect aflatoxin B1 (AFB1) in peanuts. Iron–cobalt-doped magnetic carbon (Fe-Co/NPC) was used to enhance the conductivity of the electrode and catalytic performance, providing an increased specific surface area. Gold nanoparticles (AuNPs) were used to immobilize an aptamer. And cDNA-polyacrylic acid (cDNA-PAA) nanogels served as a high-density carrier for cDNA and an active signal amplification unit, significantly increasing the charge transfer resistance (Rct) through steric hindrance and electrostatic repulsion. Unlike traditional aptasensors that relied on passive blocking agents, we designed a competitive displacement mechanism. AFB1 competed with cDNA-PAA during detection in order to bind to the aptamer, which resulted in the removal of the non-conductive complex and a substantial increase in the electrochemical signal. Under the optimal conditions, the aptasensor had a linear response range of 1–1000 ng/L and a limit of detection (LOD) of 0.3 ng/L. It displayed high specificity, reproducibility, and stability. In spiked peanut samples, the recoveries ranged from 98.04% to 100.86%. Due to its sensitivity and reliability, this aptasensor has a great determination of AFB1 in food safety applications.

## 1. Introduction

AFB1 is a mycotoxin that is primarily synthesized by Aspergillus flavus and Aspergillus parasiticus fungi [1,2]. AFB1 is the most toxic among all aflatoxins and also the most abundant [3]. It is widely detected in various crops contaminated by fungi, such as peanuts, corn, rice, soybeans, nuts, and cottonseeds [4,5,6,7,8,9]. Both humans and animals are exposed to serious health threats because of the high hepatotoxicity of AFB1, which causes acute poisoning, liver cancer, and immunosuppression [10,11,12]. It thus ranks among the most powerful natural carcinogens known [13,14].

Some of the currently existing methods of determination of AFB1 include high-performance liquid chromatography (HPLC) [15], enzyme-linked immunosorbent assay (ELISA) [16], liquid chromatography–mass spectrometry (LC-MS/MS) [17,18], quantitative fluorescence PCR (qPCR) [19], and immunochromatographic strip assays. These detection techniques are easy to apply and fast, but they typically require expensive instrumentation, trained personnel, and are not suitable for rapid on-site analysis. Biosensors have seen great improvements in the identification of AFB1, especially those that rely on electrochemical theory. These biosensors combine biorecognition features and signal transducers to provide high sensitivity and specificity in the process of AFB1 determination [20,21,22,23]. Electrochemical sensors are dependent on the characteristic biochemical interactions between recognition elements with AFB1 to produce measurable electrical signals. The research tends to target ways of improving the operation of sensors through studying ways of modifying the electrode surfaces and amplifying the signals, which should lead to enhancing the sensitivity and accuracy of the determination [24].

The Fe-Co/NPC composite materials are magnetized carbons with a porous matrix that has a large specific surface area and a huge number of pores, allowing them to contain large volumes of active ingredients and allowing the movement of ions and molecules in the reaction medium, thus significantly improving mass transfer properties [25]. In addition, the synergy of iron and cobalt along with nitrogen doping, which adjusts the electrical properties of the carbon material, does not simply increase the interaction between metal species and carbon support but efficiently prevents the aggregation of metal particles and substantially improves the catalytic active centers and magnetic characteristics of the material, which results in increased catalytic performance. Furthermore, Fe-Co/NPC has outstanding chemical stability and conductivity, so it can provide an efficient exchange of charges [26].

The PAA nanogel is an anionic hydrogel that has high flexibility and biocompatibility, is easy to prepare, and has good hydrophilicity. The structure of PAA contains abundant carboxylic acid groups, which can undergo esterification with amino-modified cDNA strands to form cDNA-PAA complexes. Owing to its low conductivity, cDNA-PAA can bind to aptamers and amplify current differences. Because of its low conductivity, these complexes can bind to aptamers and amplify the difference in current signals [27].

Many sensors use simple nanomaterial modifications without an active signal amplification strategy, resulting in a relatively narrow practical quantification range (PQR) and higher detection limits. Using the previous discussion, this section builds a sensor based on Fe-Co/NPC and cDNA-PAA, which can be applied to the identification of AFB1 in peanuts. Fe-Co/NPC enhances the specific surface area of the electrode, enabling a larger amount of aptamers to be immobilized; AuNPs are employed to immobilize the aptamers and enhance the efficiency of the electrochemical reaction, and the prepared PAA nanogel, when mixed with cDNA, yields a composite of cDNA-PAA nanogel capable of synergistically amplifying the electrochemical signal difference of a target-free and target-containing system. Its determination principle is as follows: The aptamer first hybridizes with cDNA-PAA to form an insulating barrier, resulting in a decreased current signal. Upon addition of AFB1, AFB1 binds to the aptamer with higher affinity and competitively displaces cDNA-PAA. The insulating layer detaches from the electrode’s surface, restoring electron transfer and leading to a significant increase in current. The optimal determination conditions of the designed electrochemical aptasensor displayed a detection limit of AFB1 at 0.3 ng/L and a practical determination range of 1–1000 ng/L. The recovery rate of peanut samples with different amounts of AFB1 was found to vary in the range between 98.04% and 100.86% (*n* = 3) throughout the range tested. Taken together, these findings suggest that the sensor is a reliable device for detecting AFB1, which makes it suitable to be implemented widely into the monitoring of food safety.

## 2. Materials and Methods

### 2.1. Instruments and Reagents

The source of AFB1 was Qingdao Pribolab Biotech Co., Ltd. (Qingdao, China). 2-Methylimidazole, cobalt nitrate, ferric nitrate hexahydrate Fe(NO_3_)_3_·6H_2_O (98%), potassium ferricyanide, methanol, disodium hydrogen phosphate dihydrate, and trisodium citrate dihydrate were purchased from Sinopharm Chemical Reagents Co., Ltd. (Shanghai, China). Acrylic acid (AA) was sourced from Fuchen Chemical Reagent Co., Ltd. (Tianjin, China). Triethoxysilane (APTES), glutaraldehyde, n-hexane, acetone, ethanol, and cyclohexane were purchased from Macklin Biochemical Technology Co., Ltd. (Shanghai, China). Aptamers, their complements, bovine serum albumin (BSA), and 1× TE buffer were purchased from Sangon Biotech Co., Ltd. (Shanghai, China). Peanut samples were bought at local supermarkets in Zibo city, Shandong province, China. The aptamer and its complement sequences used in [28] are given in sequence as follows:

AFB1-aptamer: 5′-HS-SH-GTTGGGCACGTGTTGTCTCTCTGTGTCTCGTGCCCTTCGCTAGGCCCACA-3′;

AFB1-complementary: 5′-CGAGACACAGAGAGACAACA-NH_2_-3′.

### 2.2. Apparatus

The evaluation of the electrochemical performance was conducted through a CHI760E electrochemical workstation. (Shanghai Chenhua Instrument Co., Ltd., Shanghai, China). Transmission electron microscopy (TEM) pictures were taken using a JSM-6700F microscope (JEOL, Tokyo, Japan). An energy-dispersive X-ray spectroscopy (EDX) experiment was conducted in a Tecnai G2 F20 system (Thermo Fisher Scientific, Waltham, MA, USA). X-ray photoelectron spectroscopy (XPS) spectra were measured using a Thermo ESACALAB 250Xi (Thermo Fisher Scientific, Waltham, MA, USA).

### 2.3. Preparation of Fe-Co/NPC

The procedure for the synthesis of the atomically dispersed Fe-Co/NPC has been reported earlier in the scientific literature. Firstly, 1.68 g of Fe(NO_3_)_3_·6H_2_O and 50 mg of Co(NO_3_)_2_·6H_2_O were dissolved in 80 mL of ultrapure water to form solution A. Simultaneously, 3.7 g of 2-methylimidazole was dissolved in 80 mL of ultrapure water to form solution B. Solutions A and B were mixed together in the same beaker and stirred during the entire process of reacting the solutions. The obtained solid was precipitated after 24 h by centrifugation, followed by repeated washing with ultrapure water, and finally freeze-dried. In order to synthesize Fe-Co/NPC, the already synthesized Fe-doped-ZIF-67 was heated to 960 °C at a ramp rate of 5 °C/min under a nitrogen (N_2_) atmosphere and maintained at this temperature for 2 h. The final Fe-Co/NPC was obtained with a yield of approximately 65% [29].

### 2.4. Preparation of AuNPs

AuNPs synthesis was done by mixing ultrapure water (48.75 mL) and chloroauric acid (1.25 mL, 0.4%) and homogenizing it using intensive stirring. Upon 7–8 min of simmering under constant stirring, a 1.0 mL sample of 1% sodium citrate solution was added rapidly to the mixture. The color of the solution slowly changed from light blue to dark blue; then, it became blue-black and eventually purple-red, finally turning into wine red. Following this, heating was stopped, and the solution was stirred further for 30 min to allow the full formation of the AuNPs [30].

### 2.5. Aptamer Immobilization via Au–S Bonding

After the deposition of AuNPs onto the Fe-Co/NPC-modified GCE, 6 μL of the 1.5 μM thiol-modified AFB1 aptamer solution was drop-coated onto the electrode’s surface and incubated for 2 h at room temperature to allow Au-S bond formation.

### 2.6. Preparation of cDNA-PAA Nanogel

The PAA nanogels were synthesized based on the protocol set by Shaik et al. Initially, a solution of 55 mL of water was agitated vigorously to mix 50 mg of SDS and 60 mg of ammonium persulfate until it was completely dissolved. Then, 50 mg of MBA and 1 mL of AA were added to the solution. The polymerization reaction was thereafter carried out by placing the mixture in a water bath kept at 80 °C. Four hours later, the PAA nanogels were formed. Two rounds of centrifugation were used to remove unreacted reagents. The resulting purified nanogels were re-suspended in 11 mL of distilled water to be used in future experiments. The 2 mL of PAA nanogel dispersion was mixed with 2 mL of the fresh EDC/NHS solution (0.2 M) to covalently connect the amino-modified cDNA through carboxyl groups at 4 °C for 1 h. After this activation process, the nanogels were centrifugally separated and washed with 2 mL of PBS buffer. At last, 100 μM cDNA (10 μL) was added to the activated nanogels and incubated at 37 °C to form the cDNA-PAA nanocomposite [31].

### 2.7. Electrode Cleaning

The glassy carbon electrode (GCE) was first polished with Al_2_O_3_ slurries (0.3 μm and 0.05 μm) until a mirror-like surface was obtained and then rinsed with ultrapure water [32]. Subsequently, the electrode was sequentially sonicated in ultrapure water, anhydrous ethanol, and ultrapure water for 3–5 min each to remove residual Al_2_O_3_ particles and organic contaminants. Electrochemical activation was then carried out in 0.5 mol/L H_2_SO_4_ by cyclic voltammetry (CV) over a potential range of −0.2 to 1.6 V for 10–15 cycles, forming a hydroxylated (-OH) coating on the electrode surface. This activation step enhanced the specific surface area and provided additional active sites for efficient aptamer immobilization [33]. Finally, the electrode was rinsed with ultrapure water to complete the pretreatment process.

### 2.8. Sample Pretreatment

Peanut samples were prepared under the multi-step pretreatment before spike recovery experiments in real samples by following these steps: Firstly, the purchased peanuts were washed, dried, and then ground into a homogenate through a blender. Then, an extraction solution was prepared by mixing 14 mL of methanol and 6 mL of ultrapure water. Thereafter, the mixture was well shaken after adding 5 g of peanut homogenate to the extraction solution and shaking it for 60 min in order to allow sufficient extraction of the analytes of interest. After the end of the mixing, the solution was centrifuged at 6000 rpm for 10 min. The filtrate was further purified in the next stage by passing it through a 0.22 µm ultrafiltration membrane. Such filtration successfully eliminated residual impurities, forming a transparent eluate that was further used in additional analytical methods.

For the determination of AFB1, the prepared electrochemical aptasensor was immersed in different concentrations of AFB1 solution and incubated for 60 min. The DPV measurements were performed using a CHI760E electrochemical workstation in a solution containing 5 mM [Fe(CN)_6_]^3−^/^4−^ in 0.1 M PBS (pH 7.0). The following parameters were applied: potential range from –0.1 to 0.6 V; pulse amplitude of 50 mV; pulse width of 50 ms; scan rate of 50 mV/s; step potential of 5 mV; quiet time of 5 s.

## 3. Results

### 3.1. Fabrication of Electrochemical Aptasensors

**Figure 1:**
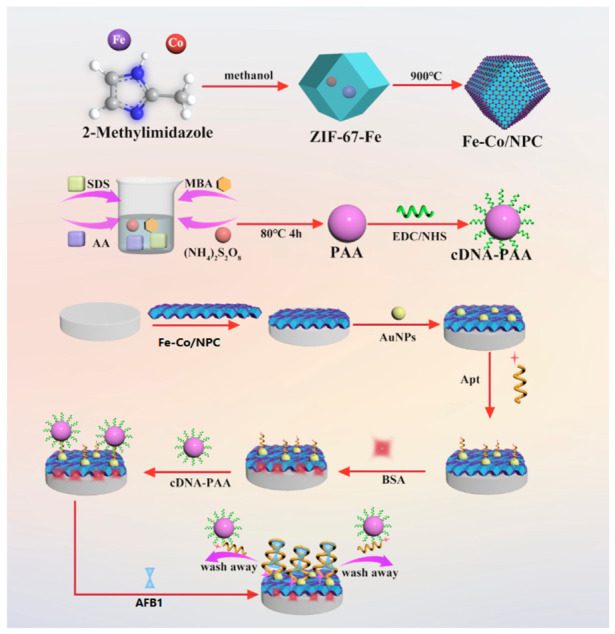
Schematic representation of the electrochemical aptasensor developed for high-sensitivity AFB1 determination.

Firstly, 7 μL of Fe-Co/NPC was carefully deposited in a uniform manner on the surface of a pretreated GCE and then air-dried at room temperature. Afterwards, 6 μL of the AuNP dispersion was dropwise added to the Fe-Co/NPC-modified electrode surface and air-dried at room temperature. The specific characteristics of AuNPs provided the points to immobilize the aptamer. Then, 6 μL of the 1.5 μM aptamer solution was deposited on the surface of the electrode, which was allowed to incubate in the air for 2 h to achieve steady immobilization of the aptamer. A 6 μL of BSA solution was used to block the remaining unoccupied non-specific areas on the electrode’s surface so as to minimize the background interference during subsequent determination. Following blocking, 6 μL of the cDNA-PAA nanogel was added dropwise to the electrode’s surface, followed by air incubation at room temperature. This point marked the end of the fabrication process of the aptasensor aimed to detect AFB1. The total assembly time is approximately 4.5 h, which is at a reasonable level. The overall electrode modification process is shown in Figure 1.

### 3.2. Characterization of Fe-Co/NPC Material

In order to increase the active surface area and achieve maximum response, the dodecagonal Fe-Co/NPC material was chosen. Fe and Co incorporation, along with its ultra-high specific surface area, offered a simple and inexpensive way of generating nanostructures on the sensor surface [34] that allowed improved binding of an aptamer. In addition, electrochemical methods of surface modification were confirmed, and the results agreed with theoretical predictions.

**Figure 2:**
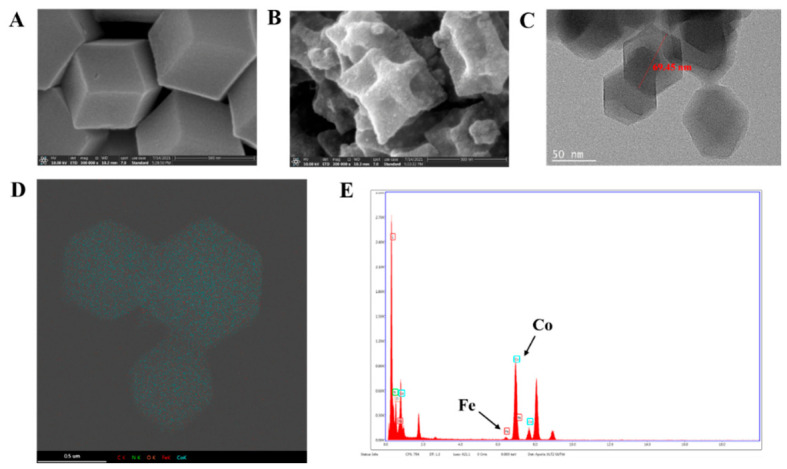
(**A**) Scanning electron microscope image of ZIF-67-Fe; (**B**) scanning electron microscope image of SA-Fe-NZ; (**C**) transmission electron microscope image of Fe-Co/NPC; (**D**,**E**) EDS image of Fe atoms present in Fe-Co/NPC.

The Co-Fe/NPC dodecagonal cage structure was used as a perfect interfacial material to connect ligands to the electrode. Figure 2A depicts a standard ZIF-67 dodecagonal framework after the addition of Fe atoms [35]. As a result of heat treatment at 960 °C under an N_2_ atmosphere within 2 h, the surface of ZIF-67-Fe collapsed to form a wrinkled cage architecture (Figure 2B) [36]. Increased aptamer adsorption and better electron transfer efficiency were achieved by the rough surface of the resultant Fe-Co/NPC substance, causing enhanced determination signals [37]. As illustrated in Figure 2C, the uniformity of the morphology of the Fe-Co/NPC material after heating was around 70 nm in size, indicating that the nanopores of about 10–20 nm were collapsed. The EDX analysis in Figure 2D,E shows that Fe-Co/NPC consists of four basic elements, Co, C, Fe, and N, with a uniform and high content of Fe and Co in the nanomaterial.

**Figure 3:**
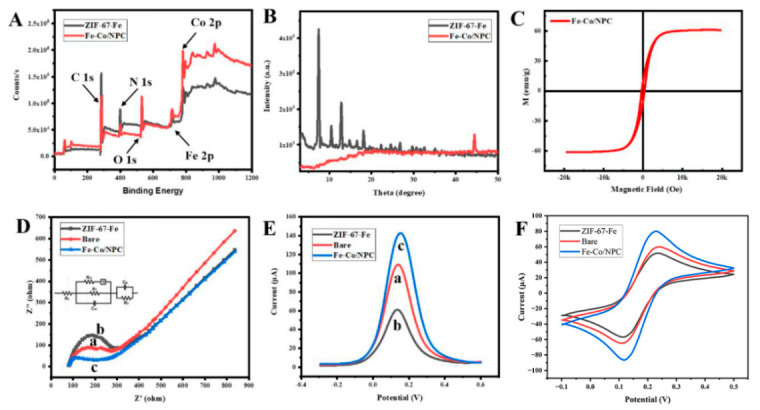
(**A**) XPS image of ZIF-67-Fe and Fe-Co/NPC; (**B**) XRD image of ZIF-67-Fe and Fe-Co/NPC; (**C**) hysteresis loop of Fe-Co/NPC; (**D**) EIS image of bare electrode, ZIF-67-Fe, and Fe-Co/NPC; (**E**) DPV image of a bare electrode (a), ZIF-67-Fe (b), and Fe-Co/NPC (c); (**F**) CV image of a bare electrode, ZIF-67-Fe, and Fe-Co/NPC.

Figure 3A shows the thermal treatment comparison of ZIF-67-Fe and Fe-Co/NPC with respect to XPS. The XPS spectra of each sample had characteristic spectral lines of C 1s (284 eV), O 1s (532 eV), N 1s (400 eV), Fe 2p (710 eV), and Co 2p (778 eV) as reported previously, which confirmed that Fe and Co were successfully incorporated into porous carbon [38]. Figure 3B depicts the X-ray diffraction (XRD) patterns of ZIF-67-Fe before and after calcination. Three major diffraction peaks at 2θ angles of 4.78°, 8.76°, and 17.88° were indexed to the (200), (101), and (400) faces of ZIF-67-Fe, and they were found to have similar peak forms. After heat treatment, these specific ZIF-67 peaks were no longer observed, which suggested that the initial structure was decomposed. New peaks appeared at 42.72° and 51.53°, corresponding to the presence of Co (111), which confirmed the synthesis of cobalt nanoparticles [38].

Figure 3C shows the hysteresis loop of Fe-Co/NPC, which was used to determine its magnetic properties. The plot showed magnetization intensity (M) on the y-axis and the external magnetic field (H) on the x-axis. The maximum value observed in the hysteresis loop of Fe-Co/NPC was about 60 emu/g, as it represented an intensely magnetized condition at the highest level of magnetic field; in contrast, ZIF-67-Fe had a value near zero, which meant it was not magnetized. These results implied that Fe-Co/NPC had a great number of magnetic properties, such as high levels of magnetization, fast response to magnetic fields, and stable performance in comparison to nearly non-magnetic ZIF-67-Fe. Moreover, the fabricated Fe-Co/NPC was found to be superparamagnetic at room temperature with no hysteresis, meaning that the composite material could easily be redispersed after the magnet is removed. Figure S1 illustrates that water was homogeneously dispersed with Fe-Co/NPC in the form of a suspension. Under the influence of a single magnet, Fe-Co/NPC was strongly attracted to the inside wall. They showed that Fe-Co/NPC had outstanding recycling ability and was reusable.

On the Nyquist plot, the electron transfer process was characterized by the semicircle at high frequencies, and it had a diameter corresponding to the Rct. As illustrated in Figure 3D, the naked electrode (curve a) had a relatively high resistance, exhibiting an Rct of 128.5 Ω. The addition of ZIF-67-Fe (curve b) resulted in a substantial increase in the size of the semicircle, which is mainly caused by the poor electrical conductivity of the ZIF-67 substance, corresponding to an Rct of 312.8 Ω. Conversely, the diameter of the semicircle decreased significantly when the electrode was modified with Fe-Co/NPC (curve c), and the Rct decreased sharply to 89.6 Ω, indicating that Fe-Co/NPC had excellent conductivity and promoted electron transfer at the electrode interface. The primary reason behind this increase in conductivity was that the exposure of Fe and Co atoms by thermal processing allowed electron transfer. Also, the differential pulse voltammetry (DPV) technique was used to assess the performance of the electron transfer on the electrode surface. Different modified electrodes were systematically examined with DPV responses (Figure 3E). Current was reduced with ZIF-67-Fe (curve b) but increased greatly with Fe-Co/NPC (curve c) when the bare electrode (curve a) was altered. This improvement was explained by the creation of electron pathways formed by Fe atoms in Fe-Co/NPC, enhancing conductivity. Figure 3F represents the CV curves of the electrode in the [Fe(CN)_6_]^3−/4−^ solution. The bare electrode (curve a) had much lower peak currents compared to the modified one using Fe-Co/NPC (curve c), with markedly higher current values. This improvement can be explained by a greater redox value of Fe-Co/NPC because of the rapid electron transfer caused by the distribution of Fe and Co atoms across the carbon network. The same observation was made based on electrochemical impedance spectroscopy (EIS) and DPV findings. The results demonstrate that the electrical signal variation of the biosensor’s construction was stronger after thermal treatment of ZIF-67-Fe, exposing the metal atoms present in Fe-Co/NPC, than in a non-thermally treated ZIF-67-Fe sample.

### 3.3. Characterization of the AuNPs, PAA Nanogel, and cDNA-PAA Material

**Figure 4:**
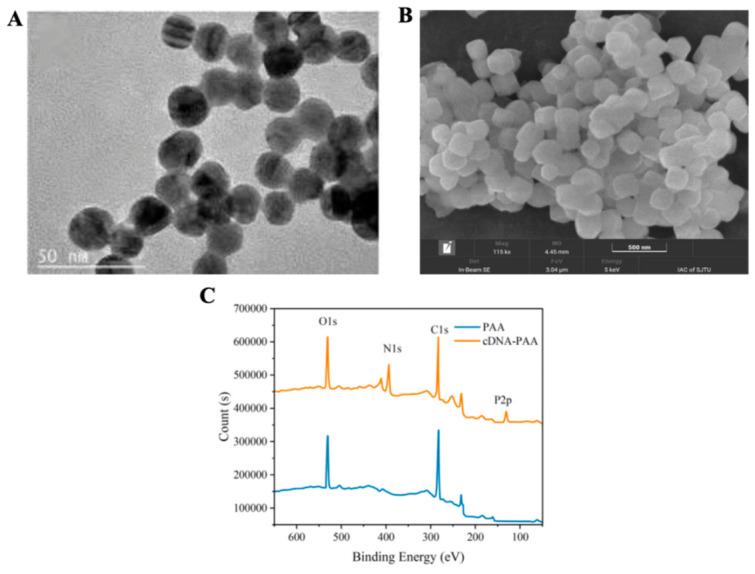
(**A**) TEM images of AuNPs; (**B**) TEM images of PAA nanogel; (**C**) XPS analysis of the cDNA-PAA nanogel.

The microstructure of the AuNPs was characterized by TEM, as shown in Figure 4A. TEM observations revealed that the AuNPs were spherical, with a diameter of approximately 15 nm. This spherical morphology provided a high surface area, which facilitated effective aptamer immobilization and thereby increased the specific surface area of the electrode.

The TEM was utilized to determine the morphology of the PAA nanogel. The PAA nanogel could be evenly spread, as seen in Figure 4B, and they were spherical in appearance, with a particle size of about 150 nm. These morphological characteristics and dimensions were consistent with those expected of PAA nanogels, thus confirming that the synthesis approach was effective and reliable.

The synthesis process of the cDNA-PAA nanocomposite was confirmed by comparing the XPS data of the elemental composition of the PAA nanogel with that of the cDNA-PAA material (Figure 4C). The XPS characterization revealed the presence of characteristic peaks at 283.6 eV and 535.3 eV, which were identified as C 1s and O 1s, respectively, in the PAA nanogel. By comparison, the cDNA-PAA material had extra peaks at 400.7 eV (N 1s) and 132.08 eV (P 2p) besides the two characteristic peaks, which were at C 1s and O 1s. These new peaks were directly linked to the existence of nitrogen (N) and phosphorus (P) in the cDNA molecules. Therefore, their presence confirmed the successful grafting of cDNA molecules onto the surface of the PAA nanogel, demonstrating the effective preparation of the cDNA-PAA nanocomposite.

### 3.4. Mechanistic Analysis of the Determination of AFB1

**Figure 5:**
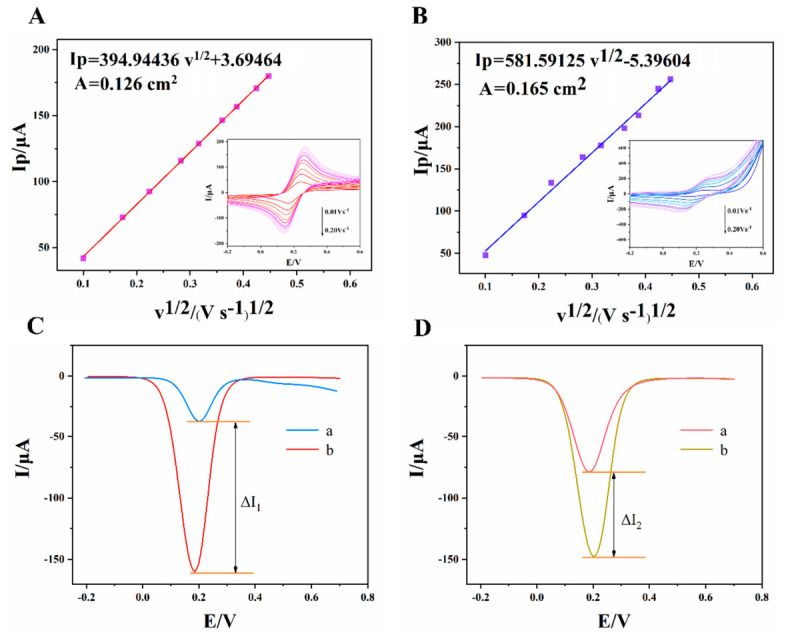
Redox peak current versus the square root of the scan rate for (**A**) bare GCE and (**B**) Fe-Co/NPC/GCE. DPV response of the sensor (**C**) with and (**D**) without the use of cDNA-PAA.

The sensor’s electrochemical response correlates with the effective surface area of the modified working electrode. Using the Randles–Sevcik equation (Ip = 2.686 × 10^5^ × n^3^/^2^ × A × D^1^/^2^ × C × ν^1^/^2^, with *n* = 1, D = 6.7 × 10^−6^ cm^2^ s^−1^), the effective area (A) was calculated as 0.126 cm^2^ for the bare GCE (Figure 5A) and 0.165 cm^2^ for the Fe-Co/NPC-modified electrode (Figure 5B). This confirms that a nanomaterial’s large specific surface area both increases the effective electrode area and facilitates electron transport [39].

DPV was then used to evaluate the sensor with and without cDNA-PAA, using the current variation (ΔI) upon AFB1 addition as the metric. ΔI_1_ (Figure 5C) was 120 μA, while ΔI_2_ (Figure 5D, where curves a and b show signals before and after AFB1 addition) was 60 μA. The larger ΔI and higher post-addition peak current both demonstrate that incorporating cDNA-PAA significantly improves sensing performance.

### 3.5. Electrochemical Characterization and Feasibility Analysis of Electrochemical Aptasensors

**Figure 6:**
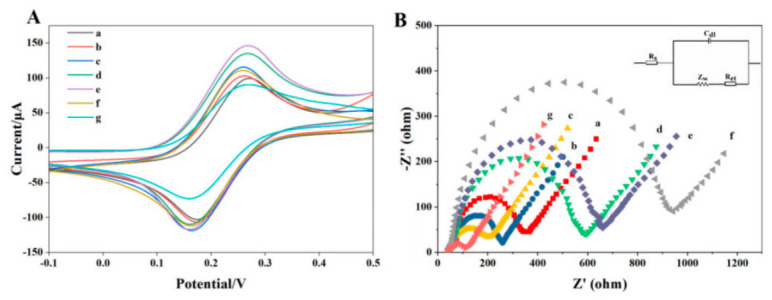
(**A**) CV and (**B**) EIS characterization of the assembly diagram of the aptasensor: (a) GCE; (b) Fe-Co/NPC/GCE; (c) AuNPs/Fe-Co/NPC/GCE; (d) Apt-AuNPs/Fe-Co/NPC/GCE; (e) BSA/Apt-AuNPs/Fe-Co/NPC/GCE; (f) cDNA-PAA/BSA/Apt-AuNPs/Fe-Co/NPC/GCE; (g) AFB1/cDNA-PAA/BSA/Apt-AuNPs/Fe-Co/NPC/GCE.

Figure 6A indicates CV curves of electrodes at various stages of modifications with [Fe(CN)_6_]^3−/4−^ solutions. The electrodes were as follows: bare GCE, Fe-Co/NPC/GCE, AuNPs/Fe-Co/NPC/GCE, Apt/AuNPs/Fe-Co/NPC/GCE, BSA/Apt/AuNPs/Fe-Co/NPC/GCE, cDNA-PAA/BSA/Apt/AuNPs/Fe-Co/NPC/GCE, and AFB1/cDNA-PAA/BSA/Apt/AuNPs/Fe-Co/NPC/GCE.

The variations of the curves can be presented as follows: In contrast with the bare GCE (curve a in Figure 6A), the electrode that was improved using Fe-Co/NPC had a much higher peak current (curve b), which was because of its remarkable electron transfer property. The peak current was even higher after the immobilization of high-conductivity AuNPs (curve c). Upon immobilizing the aptamer on the surface of the electrode, there was a reduction in the peak current (curve d), which was ascribed to electrostatic repulsion between the phosphate backbone with a negative charge of the aptamer and the negatively charged [Fe(CN)_6_]^3−/4−^ redox probe, thus preventing the flow of electrons. The addition of bovine serum albumin (BSA) to the non-specific sites subsequently resulted in a further reduction in current (curve e). As a result of the application of the cDNA-PAA nanogel to the electrode, the current sharply decreased (curve f), mainly because the nanogel was not conductive and added substantial electron transfer resistance. Lastly, upon the introduction of AFB1 (curve g), the competition between AFB1 and cDNA-PAA for aptamer binding occurred, and cDNA-PAA was released at the electrode surface. This led to a decrease in electron transfer resistance and therefore a clear recovery of the peak current.

The reliability of the CV results was verified by simultaneously using EIS to monitor the change in resistance during electrode modification. The corresponding findings are presented in Figure 6B. The result is illustrated: The bare GCE (curve a) had a semicircle on its EIS spectrum that indicated a low electron transfer resistance of 128.5 Ω. This was attributed to the clean surface of the bare glassy carbon electrode, which enabled relatively efficient electron transfer. When modified by Fe-Co/NPC and AuNPs, the Rct (charge transfer resistance) values were reduced to 89.6 Ω (curve b) and 45.2 Ω (curve g), respectively, because they had high conductivity. After the aptamer’s immobilization, the Rct increased to 185.3 Ω (curve d) as a result of the electrostatic repulsion of the aptamer and the [Fe(CN)_6_]^3−/4−^ probe. Blocking of non-specific binding sites and forming a physical barrier by BSA further increased Rct to about 210.6 Ω (curve e). Once incubated with the cDNA-PAA nanogel, Rct strongly increased to 1153 Ω (curve f), demonstrating the insulation capacity of the nanogel. Additionally, the abundant negatively charged carboxyl groups on PAA electrostatically repelled the probe, and the synergistic effect of these two factors severely impeded electron transfer. Ultimately, upon addition of AFB1, Rct dropped substantially to 55.8 Ω (curve c). AFB1 exhibited a stronger affinity for the aptamer, thereby competitively displacing the cDNA-PAA nanogel. As the insulating layer detached from the electrode surface, the electron transfer pathway was restored, leading to a substantial decrease in Rct. To summarize, the EIS outcomes were entirely similar to the CV characterization that confirmed the successful construction of the AFB1 aptasensor built in this study, with adequate performance for use in future determination applications.

### 3.6. Optimization of Test Conditions for Electrochemical Aptasensor

**Figure 7:**
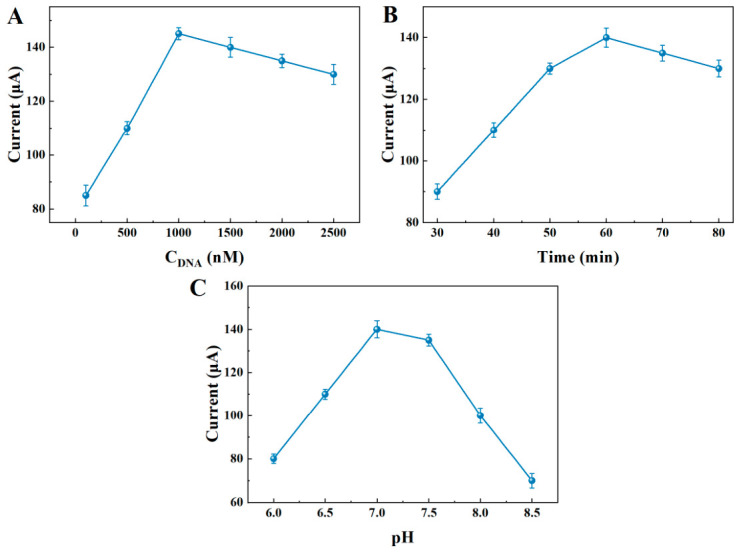
Optimization of experimental parameters: (**A**) aptamer concentration; (**B**) AFB1 incubation time with target; (**C**) pH of the solution (scan range: −0.2–0.6 V; quiet duration: 5 s). All electrochemical measurements were performed in a test solution of 5 mM [Fe(CN)_6_]^3−^/^4−^ (pH 7.0, 0.1 M KCl) with a saturated calomel reference electrode, and the error bars correspond to the standard deviations calculated from three independent measurements. The current response (I) represents the oxidation peak current obtained from DPV measurements.

To enhance the determination performance of the sensor, the optimal experimental parameters were determined in this study, including aptamer concentration, incubation time, and the pH of the testing solution. All optimization experiments were performed using differential pulse voltammetry (DPV), and the current response (I) refers to the peak current obtained from the DPV curves. The detailed optimization process was as follows:

The influence of aptamer concentration on the determination signal was examined, and the results are presented in Figure 7A. As the aptamer concentration increased from 100 nM to 1500 nM, a pronounced increase in the current response (I) was observed. However, as the concentration was further increased within the range of 1500–2500 nM, the signal gradually decreased instead. This phenomenon indicates that 1000 nM is the critical concentration at which the aptamer reaches saturated adsorption on the electrode’s surface. Beyond this concentration, excessive aptamer molecules stack and tangle on the electrode’s surface, which hinders efficient binding with the target. Therefore, 1000 nM was chosen as the optimal aptamer concentration for this experiment.

To maximize the capture efficiency of AFB1 by the aptamer, the reaction time between them was optimized at six time points: 30, 40, 50, 60, 70, and 80 min. As shown in Figure 7B, the current signal (I) increased progressively with reaction time, reaching a maximum at 60 min. Beyond this point, the signal exhibited a slight decrease but remained largely unchanged, indicating that the specific binding between the aptamer and AFB1 approaches saturation at 60 min. Therefore, 60 min was selected as the optimal reaction time.

The pH value is a key factor affecting the stability of the aptasensor, as it directly influences the activity of biological materials and the structure and performance of the aptamer. The prepared aptasensor was immersed in testing solutions with different pH values to examine their effects on the determination signal. For the pH optimization experiments, the concentration of AFB1 used was 100 ng/L, and the incubation time between the aptamer and AFB1 was 60 min. As shown in Figure 7C, when the pH ranged from 6.0 to 8.5, the current signal (I) first increased and then decreased, reaching a maximum at pH 7.0. Therefore, pH 7.0 was determined as the optimal pH for the testing solution.

### 3.7. Analytical Performance of the Aptasensor

**Figure 8:**
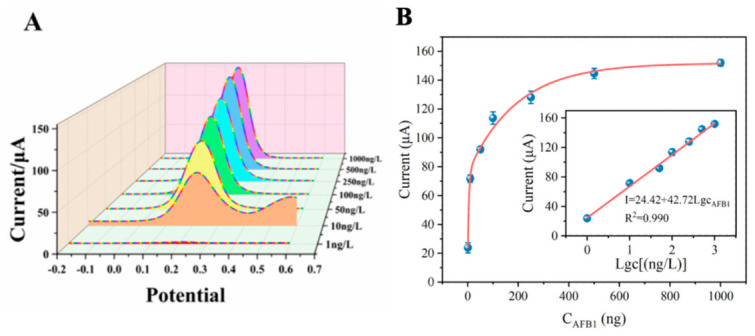
(**A**) DPV response of developed aptasensor at different concentrations of AFB1; (**B**) the standard curve of the sensor when used in detecting AFB1.

The constructed sensor’s performance in detecting AFB1 was assessed under the optimized conditions outlined above. Quantitative determination of the target was performed using seven AFB1 standard solutions with concentrations of 1, 10, 50, 100, 250, 500, and 1000 ng/L. Figure 8A shows the DPV response curves of the sensor at different AFB1 concentrations. Among them, the appearance of two redox peaks at 10 ng/L is attributed to the distinct binding states of the aptamer–AFB1 complex at low concentration, where intermediate conformational states lead to different electrochemical signals. Analysis of the corresponding data in Figure 8B indicates that the current response (I) of the sensor gradually increased with increasing AFB1 concentration. To establish the quantitative relationship between concentration and response, a calibration curve was plotted on a logarithmic scale, with the current response (I) as the ordinate and the logarithm of AFB1 concentration (1–1000 ng/L) as the abscissa (Figure 8B). The regression equation was I = 24.42 + 42.72LgcAFB1 (R^2^ = 0.990), and the limit of detection (*I_LOD_* = *I*_0_ + 3*σ*)was determined to be 0.3 ng/L.As summarized in Table 1, our sensor exhibited comparable or even superior sensitivity and a wider linear range relative to most of the existing methods.

### 3.8. Specificity, Reproducibility and Stability of the Aptasensor

**Figure 9:**
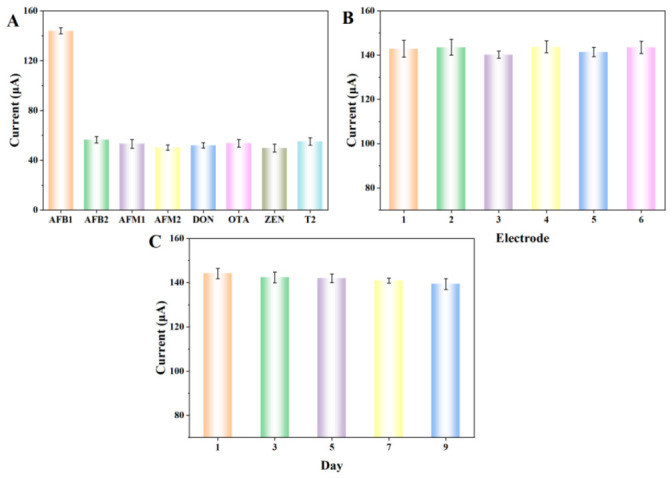
(**A**) Specificity experiment of the sensor; (**B**) reproducibility experiment of the sensor; (**C**) stability experiment of the sensor. All sensors were stored at 4 °C in a dark, dry, sealed environment (with saturated KCl solution to maintain constant humidity).

To evaluate the selectivity of the sensor, seven common mycotoxins were chosen as potential interfering substances, including AFB2, AFM1, AFM2, deoxynivalenol (DON), ochratoxin A (OTA), zearalenone (ZEN), and T-2 toxin (T2) [45], each at a concentration of 100 ng/L, which is the same as the AFB1 concentration used in the experiment. As shown in Figure 9A, a clear response signal was observed only in the presence of AFB1, while the other interfering toxins caused only relatively low levels of response. These results demonstrate that the sensor developed in this study exhibits excellent specific recognition of AFB1. To verify the reproducibility of the sensor, six groups (six parallel electrodes per group) were prepared under identical experimental conditions and used to detect AFB1 at the same concentration. Statistical analysis of the determination data showed that the sensor performed stably and consistently during parallel measurements, with a calculated relative standard deviation (RSD) of only 3.68% (Figure 9B), confirming its excellent reproducibility. Stability is a crucial factor in evaluating the practical applicability of sensors. To investigate determination stability, five sensors fabricated using the same procedure were stored at 4 °C, and their current responses (I) were measured on day 1, 3, 5, 7, and 9. As shown in Figure 9C, no significant fluctuation in I was observed within the first 5 days of storage. Even after 7 and 9 days, the electrochemical signals remained at 98.64% and 94.32% of the initial signal, respectively. These results demonstrate that the sensor possesses excellent storage stability and long-term performance for AFB1 determination.

### 3.9. Analysis of Real Sample

To assess the practical applicability of the proposed aptasensor, verification experiments were conducted using peanut samples as the test matrix. The standard addition method was applied to determine the recovery rates, thereby evaluating the reliability of the sensor in real sample matrices. The experimental results are summarized in Table 2. The recoveries of AFB1 spiked into peanut samples ranged from 98.04% to 100.86%, demonstrating values close to the theoretical expectation. These results indicate that the sensor can accurately detect the target analyte in a complex peanut matrix with minimal matrix interference. Therefore, the aptasensor developed in this study can be effectively applied to the determination and analysis of AFB1 in peanut samples, demonstrating good practical application potential.

## 4. Conclusions

An electrochemical aptasensor exhibiting ultrahigh sensitivity was designed for detecting AFB1 in peanuts. Aptamers modified with Au-S bonds served as specific recognition elements, while Fe-Co/NPC was employed to construct a signal amplification platform, thereby enhancing the intensity of the detection signals. The introduction of AuNPs played dual roles: They not only enhanced the efficiency of the electrochemical reaction but also provided stable immobilization sites for the aptamers. In addition, cDNA was combined with a PAA nanogel to form a novel signal amplifier, namely the cDNA-PAA nanogel. Due to the negative charge and low conductivity of PAA molecules, this amplifier significantly increased the current difference during determination and further optimized the signal response. After systematic optimization of the detection conditions, the sensor exhibited excellent performance: The limit of detection (LOD) for AFB1 was as low as 0.3 ng/L, with a practical determination range from 1 to 1000 ng/L. Meanwhile, the sensor demonstrated satisfactory specificity, reproducibility, and storage stability. To verify its practical applicability, the sensor was applied to the determination of AFB1 in peanut samples. The experimental results showed spiked recoveries ranging from 98.04% to 100.86% (*n* = 3). In summary, the electrochemical aptasensor developed in this study demonstrates high sensitivity and excellent stability, allowing for efficient and accurate determination of AFB1 in food samples. The sensor represents a promising platform for the determination of AFB1, with the potential to detect other mycotoxins.

## Figures and Tables

**Table 1:** Comparing several reported sensing devices for investigation of AFB1.

Working Electrode	Modified Material	Linear Range and LOD	Detection Technique	References
GCE	Thi-rGO/CS/Apt-cDNA	1–1 × 10^6^ ng/L 0.33 ng/L	ACV	[40]
GCE	Apt/MCH/cDNA/AuNPs/Co-C	1–1 × 10^6^ ng/L 0.34 ng/L	DPV	[41]
AuE	Fc-Apt1&MB-Apt2/MCH/AQ-ssDNA	10–3 × 10^3^ ng/L 4.3 ng/L	ACV	[42]
AuE	ITO/AuNPs-Apt/cDNA-MB	10–3 × 10^5^ ng/L 5 ng/L	EIS	[14]
MXene	Ti_3_C_2_T_x_	50–1 × 10^5^ ng/L 40 ng/L	EIS	[43]
AuE	AuNPs/Co-MOF	0–5 × 10^5^ ng/L 0.012 ng/L	SWV	[44]
GCE	Fe-Co/NPC/cDNA-PAA	1–1000 ng/L 0.3 ng/L	DPV	This work

Note: GCE (glassy carbon electrode); AuE (gold electrode); ACV (alternating current voltammetry); DPV (differential pulse voltammetry); EIS (electrochemical impedance spectroscopy); SWV (square wave voltammetry). Detailed material compositions are described in the original references.

**Table 2:** Recovery rates of spiked samples using the electrochemical aptasensor and HPLC in peanut samples.

Detection Methods	Sample	Added (ng/L)	Found (ng/L)	Recovery (%)	RSD (%) (*n* = 3)
Electrochemical aptasensor	peanuts	50	49.02	98.04	2.42
		100	98.94	98.94	2.35
		500	504.3	100.86	3.24
HPLC	peanuts	50	48.78	97.56	2.44
		100	99.22	99.22	1.45
		500	499.24	99.85	3.6

## Data Availability

Data are contained within the article and Supplementary Materials.

## Supplementary Materials

The following supporting information can be downloaded at https://www.mdpi.com/article/10.3390/s26144348/s1. Figure S1: Magnetic Detection of Fe-Co/NPC; Table S1: Rs and Rct of different modified electrodes during the construction and detection of AFB1.
