# Supplementary material for: Novel Electrochemical Aptasensor Based on Iron–Cobalt-Doped Magnetic Carbon and cDNA-Polyacrylic Acid for the Determination of Aflatoxin B1 in Peanuts
Source: Sensors (Basel). 2026 Jul 9;26(14):4348. doi: 10.3390/s26144348 (PMC13419315; doi:10.3390/s26144348)
Supplement: Supplementary file 1 [file sensors-26-04348-s001.zip › sensors-4312437-supplementary.pdf]

Novel Electrochemical Aptasensor Based on Iron–Cobalt-Doped Magnetic Carbon and cDNA-Polyacrylic Acid for the Determination of Aflatoxin B1 in Peanuts

Zhongyu Li [1,2,3], Zili Xia [1,2,3], Dongdong Chen [1,2,3], Yang Han [1,2,3], Heng Zhang [1,2,3], Xia Sun [1,2,3] and Wenping Zhao [1,2,3,*]

1 School of Agricultural Engineering and Food Science, Shandong University of Technology, No. 266 Xincun Xilu, Zibo 255049, China
2 Shandong Provincial Engineering Research Center of Vegetable Safety and Quality Traceability, No. 266 Xincun Xilu, Zibo 255049, China
3 Zibo City Key Laboratory of Agricultural Product Safety Traceability, No. 266 Xincun Xilu, Zibo 255049, China
* Correspondence: Wenping Zhao

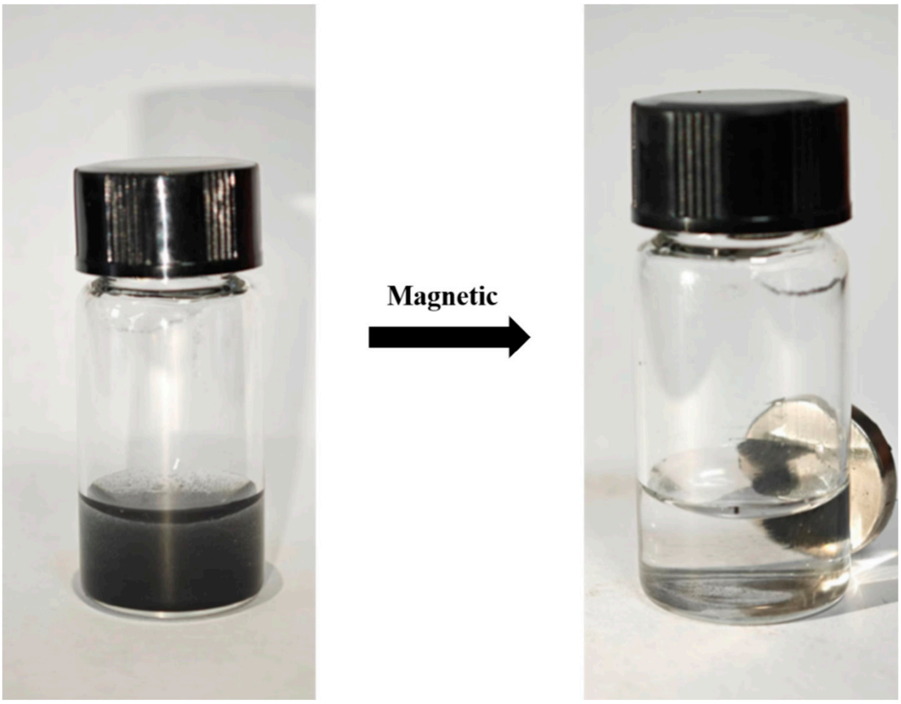

Figure S1. Magnetic Detection of Fe-Co/NPC.

Table S1. Rs and Rct of different modified electrodes during the construction and detection of AFB1.

curve	modified electrode	Rs (Ω)	**Rct (Ω)**
a	Bare GCE	34.9	**315**
b	Fe-Co/NPC/GCE	32.1	**234**
c	AFB1/cDNA-PAA/BSA/Apt/AuNPs/Fe-Co/NPC/GCE	31.6	**162**
d	Apt/AuNPs/Fe-Co/NPC/GCE	33.2	**556**
e	BSA/Apt/AuNPs/Fe-Co/NPC/GCE	32.8	**624**
f	cDNA-PAA/BSA/Apt/AuNPs/Fe-Co/NPC/GCE	34.1	**918**
g	AuNPs/Fe-Co/NPC/GCE	30.5	**87**
